# The Influence of AHI1 Variants on the Diagnosis and Treatment Outcome in Schizophrenia

**DOI:** 10.3390/ijms16022517

**Published:** 2015-01-22

**Authors:** Stefano Porcelli, Chi-Un Pae, Changsu Han, Soo-Jung Lee, Ashwin A. Patkar, Prakash S. Masand, Beatrice Balzarro, Siegfried Alberti, Diana De Ronchi, Alessandro Serretti

**Affiliations:** 1 Institute of Psychiatry, Department of Biomedical and NeuroMotor Sciences, University of Bologna, Bologna 40123, Italy; E-Mails: stefano.porcelli@yahoo.it (S.P.); beatrice.balzarro@libero.it (B.B.); siegfried.alberti@studio.unibo.it (S.A.); diana.deronchi@unibo.it (D.D.R.); alessandro.serretti@unibo.it (A.S.); 2 Department of Psychiatry, the Catholic University of Korea College of Medicine, Seoul 137701, Korea; E-Mail: crystal@catholic.ac.kr; 3 Department of Psychiatry and Behavioural Sciences, Duke University Medical Center, Durham, NC 27710, USA; E-Mail: ashwin.patkar@duke.edu; 4 Department of Psychiatry, Korea University, College of Medicine, Seoul 136701, Korea; E-Mail: hancs@korea.ac.kr; 5 Global Medical Education, New York, NY 15260, USA; E-Mail: pmasand2001@yahoo.com

**Keywords:** AHI1 gene, polymorphism, schizophrenia, antipsychotic, response

## Abstract

The present study aimed to explore whether four single nucleotide polymorphisms (SNPs) within the AHI1 gene could be associated with schizophrenia (SCZ) and whether they could predict the clinical outcomes in SCZ patients treated with antipsychotics. Four hundred twenty-six (426) in-patients with SCZ and 345 controls were genotyped for four AHI1 SNPs (rs11154801, rs7750586, rs9647635 and rs9321501). Baseline and clinical measures for SCZ patients were assessed through the Positive and Negative Syndrome Scale (PANSS). Allelic and genotypic frequencies in SCZ subjects were compared with those of controls using the χ^2^ statistics. The repeated-measure ANOVA was used for the assessment of treatment outcomes measured by PANSS changes. The case-control analysis did not show any difference in the genotypic distribution of the SNPs, while in the allelic analysis, a weak association was found between the rs9647635 A allele and SCZ. Furthermore, in the haplotype analysis, three haplotypes resulted in being associated with SCZ. On the other hand, two SNPs (rs7750586 and rs9647635) were associated with clinical improvement of negative symptoms in the allelic analysis, although in the genotypic analysis, only trends of association were found for the same SNPs. Our findings suggest a possible influence of AHI1 variants on SCZ susceptibility and antipsychotic response, particularly concerning negative symptomatology. Subsequent well-designed studies would be mandatory to confirm our results due to the methodological shortcomings of the present study.

## 1. Introduction

Schizophrenia (SCZ) is a severe and chronic psychiatric disorder that represents a major public health concern [1]. Family, twin and adoption studies show evidence for a strong genetic component in SCZ, with an estimated heritability of about 64% [2]. However, despite the evidence of the strong genetic etiology for SCZ, very little is known about the specific genes underlying such a condition.

Similarly, a genetic contribution for treatment outcome in SCZ has been continuously suggested [1,3]. Indeed, the effects of psychopharmacological treatments are mediated by biological processes, which are at least partially controlled by genetic factors [4]. Genetic research in SCZ could therefore contribute to the prediction of treatment response and side effects in individual patients, thus leading to an optimization of treatment, and could help to better understand the mechanisms of the illness, as well [4,5].

The Abelson helper integration site-1 (AHI1) locus, which is located on chromosome 6q23 and has a genomic size of 213,792 bp, encodes the protein, Jouberin, and is widely expressed in the brain (for details, see [6]). Comparative analysis of the AHI1 locus in primates proposes that the gene has undergone positive selection during the evolution of the human lineage [7]. AHI1, which is also well-known as the mouse orthologue of Jouberin, is a neuronal cytoplasmic protein that binds with huntingtin-associated protein 1 (Hap1) and, thereby, formulates a stable protein complex, which is important for maintaining the level of tyrosine kinase receptor B (TrkB), which is critical for neuronal differentiation and brain development [8,9]. Interestingly, the endogenous TrkB ligand in humans (*i.e.*, the brain-derived neurotrophic factor, BDNF) is a survival factor for parvalbumin-positive interneurons, which have been shown to be specifically altered in a series of postmortem studies in SCZ and are thought to be involved in the physiopathology of the disorder [5,10]. In addition, the literature [11,12,13,14,15,16,17,18] proposing the relationship between AHI1 and neuropsychiatric disorders, including SCZ, has been continuously increasing. Another interesting point is that AHI1 also may be possibly involved in the development of metabolic syndrome, which is important in the pharmacological management of SCZ and other neuropsychiatric disorders [19,20,21,22].

The association of AHI1 with SCZ was first reported in 2003 in an Israeli Arab family sample with high incidence of SCZ through a genome-wide linkage scan [23]. This result was subsequently replicated in a linkage analysis of a linkage peak on 6q [24] and in a fine-mapping study that identified seven markers significantly associated with SCZ [14]. The findings were subsequently replicated in an independent Icelandic case-control study [15] and in a sample of immortalized lymphoblasts of Arab Israeli SCZ patients [25]. The 6q region has been linked to SCZ in other studies, as well [15,26,27,28]. Moreover, the AHI1 gene locus has also been linked with autism, which overlaps with a SCZ haplotype [29] and is also seen in some patients with Joubert syndrome, a rare autosomal recessive disorder presenting brain dysfunction and intellectual impairment, indicating indirectly that the AHI1 gene may be involved in the crucial process of the neurodevelopmental system [30]. In our previous study [31], rs9647635 A/A was more represented in subjects with bipolar disorder as compared with major depression and healthy subjects together. rs9647635 A/A was also more represented in patients with major depression than in healthy subjects.

On the basis of the aforementioned evidence, the present paper aims to investigate whether a set of SNPs within the AHI1 gene, which were selected on the basis of previous literature data [15,17] or because they are Tag SNPs, could be associated with SCZ and to explore whether such variants could predict clinical outcome in SCZ patients naturalistically treated with antipsychotics. Taken together, the SNPs investigated allow the coverage of 4.3% of the genetic variance of the AHI1 gene, which has had 1,105 SNPs validated so far [32].

## 2. Results and Discussion

The socio-demographic features of the samples, such as gender, age and further clinical and socio-demographical variables, are reported in Table 1. For control subjects, only data on gender and age were collected. Patients and controls differ with regard to gender and age (both *p* < 0.001).

**Table 1:** Clinical and demographic characteristics of the sample. PANSS, Positive and Negative Syndrome Scale.

Variable		Schizophrenia		Controls (*n* = 345)
		Total Sample (*n* = 426)	Sample with Follow-up (*n* = 238)	
Gender	Males	198 (46.5%)	136 (57.1%)	138 (40%)
	Females	181 (42.5%)	102 (42.9%)	207 (60%)
	Missing	47 (11.0%)		
Age (years)		36.2 ± 11.73	37 ± 12.16	43.39 ± 14.05
PANSS total score	Baseline	93.91 ± 13.55	94.02 ± 13.95	
	Discharge	NA	76.63 ± 8.96	
Age at onset (years)		23.65 ± 6.6	23.28 ± 6.5	
Family history of psychiatric disorders	Yes	65 (15.2%)	38 (16.0%)	
	No	317 (74.4%)	200 (84.0%)	
	Missing	44 (10.3%)		
Suicide attempts	Yes	73 (17.1%)	46 (19.3%)	
	No	309 (72.5%)	192 (80.7%)	
	Missing	44 (10.3%)		

Data represent the mean ± the standard deviation or the number (%); NA: Not Available.

**Figure 1:**
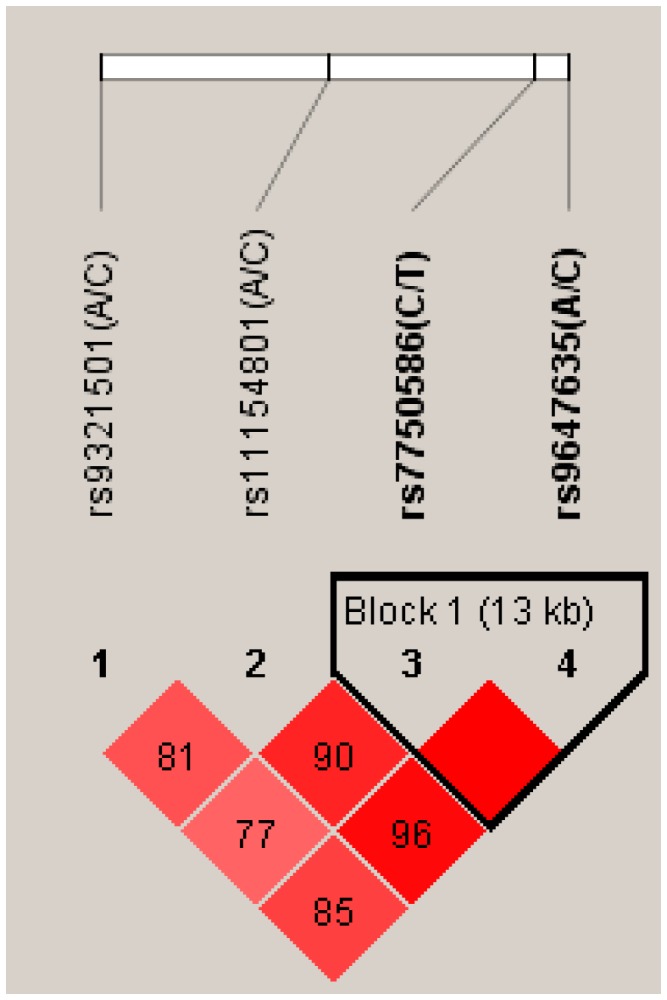
Linkage disequilibrium (LD) plot. The D' values among the SNPs investigated are shown.

**Figure 2:**
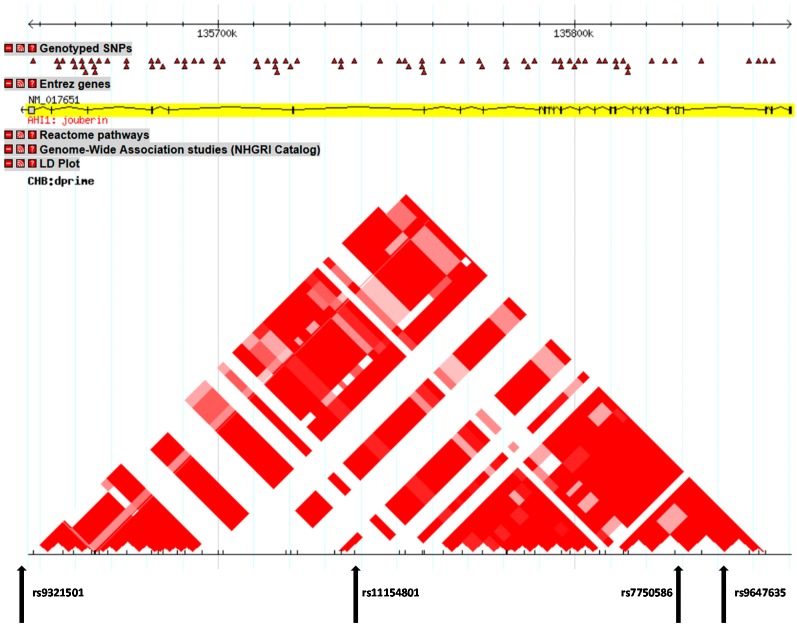
SNP positions on the AHI gene.

All of the considered SNPs were in Hardy–Weinberg equilibrium (HWE) in the whole sample (Table 2). Strong linkage disequilibrium was observed between all SNPs, particularly between rs7750586 and rs9647635, rs11154801 and rs9647635, rs11154801 and rs7750586 (for the linkage disequilibrium (LD) plot, see Figure 1. For the position of the investigated SNPs on AHI1 gene and the entire gene LD plot see Figure 2).

**Table 2:** Genotype and allele frequency of the SNPs under investigation in the present study. HWE, Hardy–Weinberg equilibrium.

SNPs	Position ^a^	HWE’s *p*-Value	Location		Schizophrenia (*n* = 426)	Controls (*n* = 345)	χ^2^	*p*-Value
**Alleles**								
rs11154801	135739355	1.0	Intron	C	671 (78.8)	542 (78.5)	0.01	0.92
	(79549)			A	181 (21.2)	148 (21.4)		
rs7750586	135827673	0.6839	Promoter	T	680 (79.8)	551 (79.8)	0.001	0.98
	(−8770)			C	172 (20.2)	139 (20.1)		
rs9647635	135841056	0.7021	Intron	A	718 (84.3)	551 (79.9)	5.10	**0.02**
	(22118)			C	134 (15.7)	139 (20.1)		
rs9321501	135641417	1.0	Intron	A	634 (74.4)	527 (76.4)	0.79	0.37
	(77487)			C	218 (25.6)	163 (23.6)		
**Genotypes**								
rs11154801	135739355	1.0	Intron	C/C	266 (62.4)	213 (61.7)	0.10	0.94
	(79549)			A/C	139 (32.6)	116 (33.6)		
				A/A	21 (4.9)	16 (4.6)		
rs7750586	135827673	0.6839	Promoter	T/T	273 (64.1)	220 (63.8)	0.10	0.95
	(−8770)			C/T	134 (31.5)	111 (32.2)		
				C/C	19 (4.5)	14 (4.0)		
rs9647635	135841056	0.7021	Intron	A/A	302 (70.9)	221 (64.1)	5.20	0.07
	(22118)			A/C	114 (26.8)	109 (31.6)		
				C/C	10 (2.3)	15 (4.3)		
rs9321501	135641417	1.0	Intron	A/A	236 (55.4)	204 (59.1)	1.13	0.57
	(77487)			A/C	162 (38.0)	119 (34.5)		
				C/C	28 (6.6)	22 (6.4)		

^a^ The relative position to the start codon is given in parenthesis. Data from [32]. In bold nominal associations.

### 2.1. Differences between Genotype and Allele Frequencies in SCZ Patients and Healthy Controls

There were no significant differences in the genotype frequencies of the investigated SNPs between the two groups, although a trend of association was found between rs9647635 and SCZ (*p* = 0.07) (Table 2). In the allelic analysis, an association between the rs9647635 A allele and SCZ was found (*p* = 0.02), although it did not survive the false discovery rate (FDR) correction. The result did not change after the inclusion of covariates in the analysis.

There were no further significant differences between genotype and allele frequencies in patients with SCZ and healthy controls. Further, in the haplotype analysis, three haplotypes resulted in being associated with SCZ (see Table 3), although one of them resulted in being very rare in the control sample (ACAC haplotype, frequency in control sample = 0.001). All of the associations survived the permutation, and they did not change after the inclusion of covariates.

**Table 3:** Haplotype analyses in the present case-control association study.

rs11154801	rs7750586	rs9647635	rs9321501	Cases Hap-Freq ^a^	Controls Hap-Freq ^a^	*p*-Value	Sim ^b^ *p*-Value	Odds Ratio
A	C	C	C	0.14	0.18	**0.04**	**0.04**	0.82 (0.62–1.09)
C	T	A	A	0.70	0.74	0.19	0.18	1 (NA)
A	C	C	A	0.01	0.02	0.40	0.40	0.77 (0.35–1.69)
C	T	A	C	0.07	0.05	0.10	0.10	1.46 (0.92–2.30)
A	T	A	C	0.02	0.01	**0.04**	**0.03**	2.45 (0.86–6.99)
A	C	A	C	0.03	0.001	**<0.001**	**<0.001**	18.85 (1.89–187.55)

^a^ Haplotype frequencies; ^b^ Simulated p value, *i.e.*, after permutation; In bold nominal associations. NA: Not applicable.

### 2.2. AHI1 Variants and Clinical Improvement in SCZ Subjects

With regard to the influence of the investigated polymorphisms on clinical improvement, in the genotype analyses, repeated-measures ANOVA did not show any association. On the other hand, in the allelic analyses, two SNPs (rs7750586 and rs9647635) were found to be associated with improvement at the negative subscale of the Positive and Negative Syndrome Scale (PANSS) (respectively *p* = 0.033 and *p* = 0.029). Interestingly, the same SNPs showed a trend of association with improvement at the PANSS negative subscale, also in the genotypic analyses (respectively *p* = 0.087 and *p* = 0.09). The results did not change after the inclusion of the covariates in the analyses. Nonetheless, no association survived the FDR correction.

The haplotype analysis did not reveal any significant association with clinical improvement in the patient sample (see Table 4).

**Table 4:** Haplotype analyses on the improvement of the PANSS total score.

rs11154801	rs7750586	rs9647635	rs9321501	Hap-Freq	*p*-Value	Sim *p*-Value
A	C	C	C	0.14	0.17	0.17
A	C	C	A	0.02	0.48	0.42
C	T	A	A	0.72	0.68	0.68
A	T	A	C	0.02	0.52	0.48
A	C	A	C	0.02	0.45	0.41
C	T	A	C	0.07	0.41	0.41

No further genotypes, alleles and haplotypes under investigation were significantly associated with clinical improvement, measured through the PANSS scale and its subscales.

The present study aimed to investigate whether four SNPs within the AHI 1gene could be associated with SCZ and whether the same variants could predict clinical outcomes in SCZ patients treated with antipsychotics.

In the case-control analysis, we failed to find any association among the SNPs investigated and the risk of SCZ. Only a weak association was found between the A allele of rs9647635 and SCZ. Nonetheless, three haplotypes of the four SNPs investigated were associated with SCZ. Torri *et al.* [13] identified 6q23.3 as a possible candidate region for SCZ and subsequently validated their finding by performing a fine mapping of the entire originally identified linkage region [14]. The best association findings were detected in a region including the AHI1, *PDE7B* and *MAP7* genes. The strongest associated SNPs within this region were rs11154801 and rs7759971. These SNPs are included in a region of approximately 500 kb encompassing the AHI1 and *BC040979* genes, which map very close to each other, within a 61-bp interval, and lie in a very high LD region that extends downstream of the 5' of AHI1 distally. Torri *et al.* [13] hypothesized that this region could contain sequences possibly having a regulatory role for both AHI1 and *PDE7B*, given that the two genes are transcribed in the opposite direction. Consistently, Slonimsky *et al.* showed an association between the rs9321501 genotype of the AHI1 gene and the AHI1 brain expression in the postmortem brain of SCZ subjects, suggesting a possible regulatory role for this SNP or for some variants linked with it [25]. Unfortunately, in the present study, we failed to replicate the previous findings, since the SNPs under investigation were not associated with the risk of developing SCZ. This could be due, at least partially, to the different ethnicity of our sample compared to previous studies, which were mainly performed on the Caucasian population. However, the results from haplotype analysis partially confirm the previous results. Nonetheless, our samples were relatively small for a genetic case-control study, and according to the power analysis, our statistical power allows detecting differences between groups equal to about an OR of 1.6, which is very uncommon for a single genetic variant in a multi-factorial disorder, such as SCZ. Thus, our negative findings may likely reflect the small power of the samples in the exam, rather than a real absence of the effects of the variants in exam. Therefore, further studies with larger samples or a meta-analysis are needed to clarify the role of the AHI1 gene in the determination of the risk for SCZ.

Further, in the present study, we failed to find any effect of the SNPs in the exam on the antipsychotic response measured by the PANSS scale. However, in the allelic analyses, we found that two SNPs, rs7750586 and rs9647635, were associated with the improvement at the negative subscale of the PANSS. Although these associations were weak and did not survive the FDR correction, we have to consider that our study is the first one investigating the relationship among AHI1 variants and antipsychotic response in SCZ patients. Therefore, our results support further investigations of this gene in the antipsychotic pharmacogenetic field, particularly concerning negative symptomatology. This could be very interesting, since the negative symptoms are known to be less responsive to antipsychotic treatments; thus, the detection of the predictor of the response for these deleterious symptoms may be useful from a clinical point of view, since it could allow planning specific treatments for those patients who respond worse to antipsychotic drugs. Interestingly, rs7750586 was reported to be associated with the risk of SCZ in three previous studies [14,15,17]; particularly, in a large case-control association study performed on two different samples from Germany and Spain, rs7750586 was associated with SCZ, both in the genotype and in the allelic analyses [17]. Contrary to the findings mentioned above, we did not observe any significant association between rs7750586 and SCZ susceptibility. On the other hand, we observed an association between this SNP and improvement in negative symptomatology, as well as for rs9647635. Considering the relevance of negative symptoms for SCZ patients’ outcome, our results may partially support the importance of AHI1 gene variants in the pathogenesis of SCZ itself and, particularly, in the determination of the antipsychotic response.

There are many reasons that could explain the discrepancy of our results compared to literature data, and these represent the main limitations of the present work. Firstly, candidate gene studies, such as the present one, are associated with a high likelihood of false positive findings [33]. This is particularly true for studies with relatively small sample sizes, like our one, which may have a quite low statistical power. Further replications in independent samples are needed to confirm our results. A further concern is related to the use of different antipsychotics with different mechanisms of action for each cohort of patients, which do not allow one to draw definitive conclusions with regard to the influence of the SNPs under investigation upon specific or classes of medications. However, our decision to include patients treated with different drugs could have the advantage of being closer to “real-world” clinical practice. Additionally, our diagnosis of psychiatric disorder was based on current DSM-IV criteria and also complemented by a structured interview with Mini-International Neuropsychiatric Interview (MINI). However, with this instrument, there is neither the ability to rule out all possibilities that patients with SCZ could switch in the future to a diagnosis of bipolar disorders, nor to exclude a possible switch to schizoaffective disorder. Such issues are important for genetic studies, as shown by recent findings suggesting that bipolar disorder and SCZ could share many important risk genes [2]. Furthermore, we asked healthy controls to report only known psychiatric disorders among first- and second-degree relatives, thus limiting the possibility to detect whether sub-threshold or untreated psychiatric disorders among family members of healthy control subjects could exist. Moreover, the healthy control sample differed from the patient sample, both for gender and age, and this could reflect a sort of selection bias, because volunteers were more often female and being of an older age than the patients. However, these differences may be just due to a better inclination of this kind of subject in the Korean population to participate as a volunteer in research studies, rather than to other possible selection biases. A further possible limitation of the present study could be imputed to the incomplete coverage of genes under investigation. Finally, sample heterogeneity could not be completely excluded, although our subjects were all native Koreans, which are considered to be genetically homogenous [26].

## 3. Methods

### 3.1. Subjects

The sample under investigation in the present study comprised 426 in-patients with SCZ who were consecutively recruited at the Department of Psychiatry of the Catholic University of Korea College of Medicine, Seoul, Korea. Patients were eligible for inclusion if they had a documented clinical diagnosis of SCZ according to the DSM-IV criteria, as assessed by the Mini-International Neuropsychiatric Interview (MINI) [34]. Patients were excluded if they currently had comorbid psychiatric disorders other than SCZ (e.g., bipolar disorder, substance-related disorders, *etc.*) and unstable medical/neurological conditions. Follow-up evaluations were available for 238 SCZ patients; thus, this subsample was used to perform pharmacogenetic analyses. A healthy control of 345 Koreans underwent the same assessment as did psychiatric patients for the exclusion of possible psychiatric disorders, and they were also asked for the presence of any known psychiatric disorder in first- and second-degree relatives. All SCZ patients admitted to the hospital were assessed by trained psychiatrists for the severity of illness at baseline and at discharge by the administration of psychometric questionnaires. In particular, SCZ severity was assessed by the administration of the Positive and Negative Symptoms Scale (PANSS) [35]. The raters were trained with specific instruments with good inter-rater reliability (*k* > 0.8). Additionally, the following clinical and demographic variables were recorded for all of the patients recruited: gender, age, age at onset, family history of psychiatric disorders, lifetime suicide attempts, duration of admission, drugs at discharge and concomitant anxiolytics. The study protocol was approved by the institutional review board (Approval Number HC10TISI0031).

### 3.2. Statistical Analyses

Traditional statistical analyses were performed using the “Statistica” package (StatSoft I. STATISTICA 7.0 per Windows: StatSoft, Inc., Tulsa, OK, USA, 1984–2004), while the test for associations using multi-marker haplotypes was performed using the statistic environment “R cran”, package “haplo.score” (http://cran.r-project.org/). The main outcome measures of the present study were: (1) differences among genotypic and allelic frequencies in patients with SCZ compared with controls; and (2) the possible influence of the 4 SNPs under investigation on clinical improvement, as measured with the PANSS total score in SCZ patients. Further sub-outcomes of interest included improvement on the PANSS subscale scores (positive, negative and general subscales) in SCZ patients. Although the genotype analysis was the primary analysis, we decided to perform also allelic and haplotype analyses in order to better elucidate the associations among the phenotypes of interest and the genetic variants investigated. Indeed, in this way, we better understand the role of a single allele, as well as the potential additive effects among the genetic variants investigated.

Differences in the allelic and genetic frequencies between healthy subjects and patients were calculated using the χ^2^ statistics (or Fisher’s exact test). The repeated measures ANOVA was used to investigate the association among genotypes and the variation over time of the PANSS total score. In the case of positive findings, the following clinical variables were added as covariates in order to investigate possible confounders: gender, age, age at onset, family history of psychiatric disorders, lifetime suicide attempts, duration of admission, medications at discharge and concomitant anxiolytics. Haploview 4.2 (Daly Lab at the Broad Institute, Cambridge, MA, USA) was used to generate a linkage disequilibrium (LD) map and to test for Hardy–Weinberg equilibrium (HWE) [36]. Gender, age, age at onset, family history of psychiatric disorders, lifetime suicide attempts, duration of admission, drugs at discharge and concomitant anxiolytics were added as covariates in the case of positive findings. Permutations (*n* = 100.000) were performed to estimate the global significance of the results for all haplotype analysis. Only haplotypes with >1% prevalence in the patient sample were included in the analysis because of the relatively small size of our sample, which did not allow for the performance of an adequate analysis on rare haplotypes. All *p*-values were 2-tailed. In order to reduce the likelihood of false positive findings, statistical significance was set at the level of 0.01, as calculated by the means of the false discovery rate (FDR), which allow for a correction of multiple testing without being as conservative as the Bonferroni correction [37]. G-Power (http://www.psycho.uni-duesseldorf.de/aap/projects/gpower/) was employed to perform the power analysis. With these parameters (*p* = 0.01), we had a sufficient power (0.80) to detect small-medium effect sizes (*w* = 0.125), which, as an example, corresponded to an odds ratio (OR) of 1.6 between the group of patients and the group of controls.

### 3.3. Genotyping

Genomic DNA was extracted from blood by standard methods and quantified. High-throughput genotyping using a pyrosequencer (Biotage AB, Uppsala, Sweden) was used for genotyping the four SNPs (rs11154801, rs7750586, rs9647635, rs9321501) of AHI1 under investigation. PCR primers (Bioneer, Daejeon, Korea) and sequencing primers (Bioneer) used for the pyrosequencing assay were designed by using the Pyrosequencing Assay Design Software v.1 (Biotage), and 1 primer of each primer set was biotinylated.

## 4. Conclusions

In conclusion, our study partially confirms a possible role of AHI1 gene variability in SKZ susceptibility and preliminarily suggests an association between AHI1 gene variants and clinical outcomes in SCZ patients. In particular, rs7750586 and rs9647635 seem to be the most promising candidate polymorphisms. However, further research is needed to confirm and extend our findings, in particular subsequent well-designed, adequately-powered pharmacogenetic research will be necessary (e.g., covering a huge number of SNPs, expanding to larger portions of the gene, prospective design and unified treatment with the same antipsychotics).
